# Aggressive treatment of metastatic squamous cell carcinoma of the rectum to the liver: a case report and a brief review of the literature

**DOI:** 10.1186/1477-7819-4-49

**Published:** 2006-08-08

**Authors:** Theodosios K Theodosopoulos, Athanasios D Marinis, Nikolaos A Dafnios, John G Vassiliou, Lazaros D Samanides, Eleni E Carvounis, Vassilios E Smyrniotis

**Affiliations:** 1 Second Department of Surgery, Areteion University Hospital, Athens Medical School, University of Athens, 76 Vasilisis Sofias av., 11528, Athens, Greece; 2 Department of Pathology, Areteion University Hospital, Athens Medical School, University of Athens, 76 Vasilisis Sofias av., 11528, Athens, Greece

## Abstract

**Background:**

Rectal squamous cell carcinoma (SCC) is a rare tumor. The incidence of this malignancy has been reported to be 0.25 to 1 per 1000 colorectal carcinomas. From a review of the English literature 55 cases of SCC of the rectum have been published. In this study we report a rectal metastatic SCC to the liver, discussing the efficacy of aggressive adjuvant and neo-adjuvant therapies on survival and prognosis.

**Case presentation:**

A 39-year-old female patient with a pure SCC of the rectum diagnosed endoscopically is presented. The patient underwent initially neoadjuvant chemo-radiotherapy and then abdominoperineal resection with concomitant bilateral oophorectomy and hysterectomy, followed by adjuvant chemo-radiotherapy. Five months after the initial operation liver metastasis was demonstrated and a liver resection was carried out, followed by adjuvant chemotherapy. Eighteen months after the initial operation the patient is alive.

**Conclusion:**

Although prognosis of rectal SCC is worse than that of adenocarcinoma, an aggressive therapeutic approach with surgery as the primary treatment, followed by combined neo- and adjuvant chemo-radiotherapy, may be necessary in order to improve survival and prognosis.

## Background

The squamous cell carcinoma (SCC) of the rectum is a rare tumor with a not yet clarified pathogenesis mechanism and a rather aggressive clinical behavior. It has a slight female predominance and prognosis worse than that of adenocarcinoma. Radical surgery is the cornerstone of treatment. However, the role of chemotherapy and radiation in the pre- and postoperative period remains to be elucidated. In the present study a case of a metastatic pure SCC of the rectum to the liver is presented. Aggressive surgical treatment as well as combined neoadjuvant and adjuvant chemotherapy and radiation is described, suggesting a possible positive survival and prognostic effect.

## Case presentation

A 39-year-old female patient was admitted to our Medical Center's surgical department with a 2 month history of altered bowel habits, perianal pain and rectal bleeding. Colonoscopy demonstrated a circumferentially developing ulcerated tumor 8 cm above the dentate line, extending 4 cm proximally. The colonoscope was advanced past the tumor, performing a total colonoscopy, while biopsies from the lesion were obtained and examined histologically, revealing a non-keratinized squamous cell carcinoma of the rectum. Abdominal computed tomography and chest radiograph were negative for metastatic disease. However, a transanal endoscopic ultrasound demonstrated the spread of the tumor beyond bowel's wall in the perirectal space, necessitating institution of neoadjuvant therapy. The patient underwent 1 session of neoadjuvant chemotherapy with 5-Fluorouracil (1000 mg/m^2^/d) plus Mitomycin and 5 sessions of radiotherapy (totally 20 Gy). Two days after the last session of radiotherapy a laparotomy was performed. Intraoperative findings included invasion of the posterior vaginal wall from the tumor and an abdominoperineal resection and a concomitant total hysterectomy-oophorectomy were carried out. Histology reported the presence of two ulcerated lesions (1 × 2 cm and 2 × 4 cm) in middle and lower rectum both squamous cell carcinoma of the rectum, invading the bowel wall and the adjacent vaginal wall. The surgical specimen contained 15 lymph nodes, all negative for metastatic disease (T4 N0 M0, stage IIb) and the resected margins were free of tumor. Postoperatively, adjuvant chemotherapy (1 session of 5-FU plus Mitomycin) and 23 sessions of radiotherapy (totally 45 Gy) were administered.

**Figure 1:**
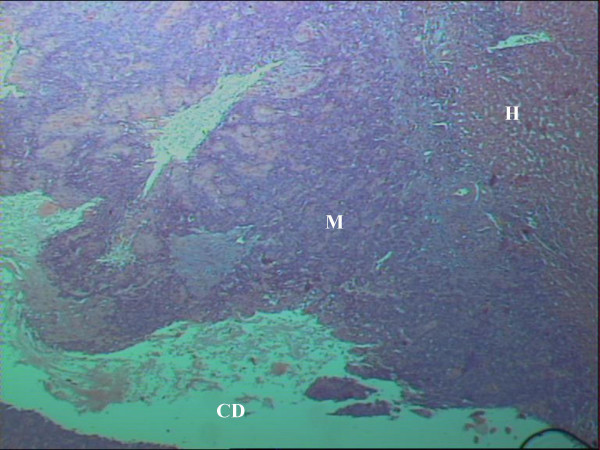
Rectal SCC metastatic to the Liver. The hepatic parenchyma, a metastatic deposit and a cystic degeneration of the tumor are shown (H&E stain, × 25).

**Figure 2:**
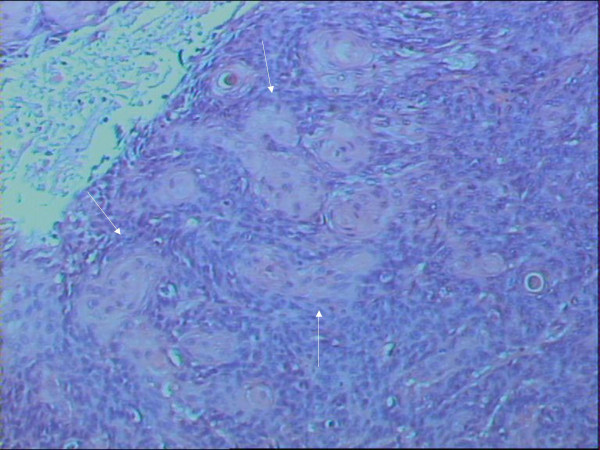
Rectal SCC metastatic to the Liver. A high power view of the tumor, showing squamous differentiation (H&E stain, × 100).

Five months after tumor resection, liver metastasis was demonstrated in a follow-up abdominal computed tomography and a central hepatectomy was performed. Histology confirmed the presence of a metastatic rectal squamous cell carcinoma to the liver (Figure 1, 2). Adjuvant chemotherapy consisting of 6 sessions of 5-FU plus Cisplatin (100 mg/m^2^/d) was applied. Fortunately, the patient had an uneventful postoperative course and is alive 18 months after.

## Discussion

Since the first report of rectal SCC by Raiford in 1933 [1], few cases have been reported in the literature. A PubMed search revealed a total of 55 case reports published since 1943 in the English literature (Table 1). Surprisingly and in contrast to the English reports, a Russian paper in 1984 reviews the clinical manifestations of 107 cases of SCC of the rectum [8].

**Table 1:** Cases of SCC of the rectumreported in the literature.

**Author**	**Date**	**Age**	**Gender**	**Treatment**	**Outcome**
Catell & Williams [2]	1943	63	M	LAR	Alive 3,5 years
Wiener et al [3]	1962	52	F	APR	Died at 1 year
Comer et al [4]	1971	34	F	APR	Alive at 13 years
''		N/A	N/A	N/A	N/A
Williams et al [5]	1979	N/A	N/A	N/A	N/A
Lasser et al [6]	1980	65	F	N/A	Alive at 3 years
''		48	F	N/A	Alive 8 months
''		54	M	N/A	Alive 17 months
Vezeridis et al [7]	1983	56	M	APR	Intraoperative death
"		44	M	APR	Died on 9 po day
"		61	F	CT	Died at 4 months
"		66	F	CT & RT	Died at 15 months
"		62	F	APR	Died at 13 months
Mel'nikov et al [8]	1984			107 CASES REPORTED	
Lafreniere & Ketcham [9]	1985	N/A	N/A	N/A	Alive at 2 years
Piggot & Williams [10]	1987	N/A	N/A	APR	Alive 1 year
Woods [11]	1987	N/A	N/A	N/A	N/A
Singh & Wong [12]	1987	N/A	N/A	N/A	N/A
Prener & Nielsen [13]	1988	N/A	N/A	16 CASES REPORTED	N/A
Schneider et al [14]	1992	43	F	Surgery & RT	Alive 7 months
"		44	M	Surgery & RT	N/A
"		69	F	Surgery & RT	Alive 6 months
Petrelli et al [15]	1996	62	M	APR	-
Martinez et al [16]	1996	40	M	LAR & RT & CT	Alive 18 months
Copur et al [17]	2001	N/A	N/A	APR & CT & RT	N/A
Frizelle et al [18]	2001			9 CASES REPORTED	
Gelas et al [19]	2002	47	F	APR & RT	Alive at 16 years
"		63	M	APR & CT & RT	Died at 14 months
"		70	F	APR & RT	Died at 18 months
"		93	M	RT	Died at 4 months
"		45	F	LAR & CT & RT	Alive at 6 months
"		43	F	LAR & CT & RT	Alive at 2 years
Anagnostopoulos et al [20]	2005	75	M	APR & CT	Alive at 14 months

LAR = Low Anterior Resection; APR = Abdominoperineal Resection; CT = Chemotherapy; RT = Radiation Therapy; F = Female; M = Male ; N/A = not available.

Rectal SCC is a rare tumor. The incidence of this malignancy has been reported to be 0.25 to 1 per 1000 colorectal carcinomas [4,5,7,21]. In 1979, Williams *et al*. [5] described three criteria that are useful today in diagnosing a primary colorectal SCC: first, metastases from other sites (e.g. lung SCC) must be ruled out; second, a squamous-cell-lined fistula must not involve the affected bowel and third, a SCC of the anus extending to the rectum must be excluded.

It seems that the pathogenesis of this malignancy is not yet clear and many theories exist [18-20]: the pluripotent stem cell theory supports that squamous cell carcinomas arise within poorly differentiated tumors [22]; the theory of proliferation of uncommitted basal cells into squamous cells, which undergo malignant transformation due to chronic injury of the glandular epithelium [23]; the theory of chronic irritation from ulcerative colitis [24], radiation exposure [25] and human papilloma virus [26], resulting in squamous metaplasia of the epithelium; and lastly, the theory of squamous differentiation of adenomas and adenocarcinomas.

Clinical manifestations of SCC of the rectum are similar to those of adenocarcinoma: change in bowel habits, pain, tenesmus and rectal bleeding, anorexia and weight loss. The natural history of this malignancy is not clearly defined due to the rarity of the disease. From data presented in Table 1, SCC of the rectum has a slight female predominance and the mean age at the time of diagnosis is 54.2 years in women and 59.4 years in men. Prognosis is worse than that of adenocarcinoma and there are some features that probably predict a poor outcome: nodal involvement, small cell or undifferentiated histological characteristics, ulcerated or annular carcinomas, grade 3 or 4 and stage IV disease [18-20].

Immunochemistry is helpful in diagnosing SCC using several markers (cytokeratins AE1/AE3, 34BE12, CK5, involucrin, etc) in order to differentiate it from other small cell undifferentiated tumors [20].

The definite therapy is surgical resection of the affected rectum, either by low anterior resection (LAR) or by abdominoperineal resection (APR), depending on the location of the tumor and the patient's preference. An informed consent and a detailed discussion of complications, adjuvant therapy, recurrence and survival-death statistics are definite prerequisites before any operation. Copur *et al*. [17] propose the use of the squamous cell carcinoma antigen (SCC Ag) as a tumor marker for following up patients. In his study the SCC Ag was elevated at the time of recurrence and showed a partial response to combination chemotherapy, with a decrease in the serum level.

The role of neoadjuvant radiotherapy (local control of the disease and downstaging) or adjuvant chemo and/or radiotherapy remains to be elucidated together with understanding the natural history of the disease. It seems that adjuvant therapy can be used in patients with nodal involvement, poorly differentiated cancers and metastatic disease without any proven effectiveness in outcome. Juturi *et al *[27] report that adjuvant combined chemotherapy reduced the size of liver metastasis in a patient with a SCC of the descending colon. However, large randomized prospective trials are needed to measure efficacy and benefits on survival and prognosis of complementary therapies.

## Conclusion

SCC of the rectum is a rare and aggressive malignancy with a similar clinical presentation pattern, but a worse prognosis than that of adenocarcinoma, especially if positive nodes are involved or poor histological features are reported. Surgical resection is the treatment of choice, although radiation and chemotherapy can be useful in treating node-positive patients and advanced disease. It is possible and remains to be studied that aggressive combined chemo-radiotherapy is required pre- and postoperatively in order to control this aggressive malignancy.

## Competing interests

The author(s) declare that they have no competing interests.

## Authors' contributions

**TT, LS **and **ND **carried out the surgical procedures and contributed to the design of the study; **TT **and **AM **gathered the data, drafted the manuscript and critically revised it; **EC **performed the histological analysis of all surgical specimens and provided histological sections as figures for the manuscript; **JV **and **VS **revised and finally approved the manuscript for been published.

All authors read and approved the manuscript.
